# Emerging Approaches to Understanding Microvascular Endothelial Heterogeneity: A Roadmap for Developing Anti-Inflammatory Therapeutics

**DOI:** 10.3390/ijms22157770

**Published:** 2021-07-21

**Authors:** Qingliang Yang, Harshani Wijerathne, Jordan C. Langston, Mohammad F. Kiani, Laurie E. Kilpatrick

**Affiliations:** 1 Department of Mechanical Engineering, Temple University, Philadelphia, PA 19122, USA; tug44932@temple.edu (Q.Y.); mkiani@temple.edu (M.F.K.); 2 Department of Chemistry, University of Kansas, Lawrence, KS 66045, USA; harshani@ku.edu; 3 Department of Bioengineering, Temple University, Philadelphia, PA 19122, USA; tuj27061@temple.edu; 4 Department of Radiation Oncology, Lewis Katz School of Medicine, Temple University, Philadelphia, PA 19140, USA; 5 Center for Inflammation, Clinical and Translational Lung Research, Department of Thoracic Medicine and Surgery, Lewis Katz School of Medicine, Temple University, Philadelphia, PA 19140, USA

**Keywords:** microvascular endothelial cells, heterogeneity, inflammation, endothelial barrier permeability, leukocytes, transmigration, microphysiological systems, bMFA, sepsis, protein kinase Cδ

## Abstract

The endothelium is the inner layer of all blood vessels and it regulates hemostasis. It also plays an active role in the regulation of the systemic inflammatory response. Systemic inflammatory disease often results in alterations in vascular endothelium barrier function, increased permeability, excessive leukocyte trafficking, and reactive oxygen species production, leading to organ damage. Therapeutics targeting endothelium inflammation are urgently needed, but strong concerns regarding the level of phenotypic heterogeneity of microvascular endothelial cells between different organs and species have been expressed. Microvascular endothelial cell heterogeneity in different organs and organ-specific variations in endothelial cell structure and function are regulated by intrinsic signals that are differentially expressed across organs and species; a result of this is that neutrophil recruitment to discrete organs may be regulated differently. In this review, we will discuss the morphological and functional variations in differently originated microvascular endothelia and discuss how these variances affect systemic function in response to inflammation. We will review emerging in vivo and in vitro models and techniques, including microphysiological devices, proteomics, and RNA sequencing used to study the cellular and molecular heterogeneity of endothelia from different organs. A better understanding of microvascular endothelial cell heterogeneity will provide a roadmap for developing novel therapeutics to target the endothelium.

## 1. Introduction

### Structure/Function of the Endothelium

The endothelium is the innermost cell layer of blood and lymphatic vessels, from the largest arteries to the smallest capillaries, forming a hierarchical network. Complementing the function of the blood vasculature, the lymphatic vasculature continuously removes excess interstitial fluid that leaks from blood vasculature and returns it into the blood flow to maintain tissue fluid homeostasis. The lymphatic endothelium also plays a major role in immune reactions, as reviewed elsewhere [1,2]. The blood endothelium serves as an interface between circulation and surrounding tissue, with most intercellular communication occurring within the microvasculature composed of arterioles, capillaries, and venules [3,4]. The endothelium is not a passive element; rather, with its secretory, synthetic, metabolic, immunologic, and surface expression functions, the endothelium plays a critical role in regulating multiple physiological processes, including the control of vascular tone, angiogenesis, and barrier permeability [5,6,7,8].

In the quiescent state, endothelial cells (EC) maintain blood fluidity, regulate vascular permeability, and maintain limited interactions with circulating leukocytes. During acute inflammation in pathological conditions, such as sepsis, COVID19, or radiation exposure, a cascade of inflammatory events can alter the vascular endothelium phenotype, resulting in coagulation, increased barrier permeability, and leukocyte trafficking into critical organs [3,4,9,10,11]. It is now well recognized that the endothelium has a critical role in the inflammatory response and plays a key role in proinflammatory mediator production, coagulation, leukocyte trafficking, and repair of damaged organs, making the endothelium a critical participant in most inflammatory disease states and a potential therapeutic target. While all EC across the vasculature in different organs and in different species share common properties, they can exhibit several phenotypes in different organs, and even vascular beds within the same organ [3,4,6]. This EC heterogeneity is expressed as diverse cell morphology, function, and patterns of gene expression [3,4]. Despite the complexity of heterogeneity, EC provide a potential for targeted drug delivery to specific EC surface markers in a variety of diseases, such as cancer and pulmonary disease [10,11,12,13,14]. This review will focus on the heterogeneity of endothelial cells, with a particular emphasis on the role of the endothelium in two types of acute inflammatory diseases which target lung endothelia (sepsis and COVID-19), the emerging technologies to study the cellular and molecular heterogeneity of endothelium, and novel microphysiological systems employing both human and animal cells to increase translatability for drug development.

## 2. Endothelium Structure and Heterogeneity

### 2.1. Endothelium Heterogeneity across Organs

During development, EC originate from mesoderm precursors to form a primitive vasculature (vasculogenesis) [15,16]. Maturation of the vasculature proceeds with the formation of new vessels by EC sprouting and splitting through angiogenesis. In response to epigenetic signals and microenvironmental cues, the EC differentiate into arteries, veins, capillaries, and lymphatic vessels [3,6,17,18]. Key regulators involved in vasculogenesis and angiogenesis are VEGF (vascular endothelial growth factor), Ang ½ (angiopoietin), Tie-2, and TGF-β (transforming growth factor-β) [19]. Members of the ETS and FOX transcription factor families have been implicated in early vascular development; however, the epigenetic signals required for EC differentiation remain to be fully delineated [20]. EC are capable of sensing and responding to the extracellular microenvironment stimuli, including biomechanical (e.g., shear force, pressure and cyclical strain) and biochemical stimulus (e.g., growth factors, hormones, cytokines, chemokines, nitric oxide, oxygen, and reactive oxygen species). EC phenotypes display marked spatial and temporal organ-specific heterogeneity corresponding to their responses to these stimuli [6]. The development of omics technology has revealed molecular signatures of tissue-specific microvascular endothelial cells, as discussed in Section 4. A DNA microarray study compared the transcriptional profiles between arterial and venous EC, and between macrovascular and microvascular EC, demonstrating the epigenetic signature still exists after multiple passaging of EC [21].

The morphological heterogeneity of endothelium is coupled to its functional heterogeneity. Connected by tight junctions, the endothelia of arteries and veins form a continuous and uninterrupted interface, surrounded by basement membranes and layers of smooth muscle cells. In arteries and arterioles, EC align and elongate parallel to the direction of blood flow and regulate vascular tone. In contrast, EC in veins and venules are mostly polygonal instead of elongated in shape, lack specific orientation, and are generally more permeable than arteries with post-capillary venules serving as the primary sites of leukocyte extravasation during inflammation [22]. These characteristics are closely correlated to differences in both hemodynamic environments and the functional properties of vessels [23]. As the smallest blood vessels, with the diameter of 5 to 10 µm formed by a single layer of EC, capillaries are the primary site of substance exchange with the surrounding tissues. Capillary endothelia can be classified as continuous, fenestrated, or discontinuous (Table 1). Continuous capillaries permit the diffusion of water, small molecules, and lipid-soluble materials, but not plasma proteins, into the surrounding tissues and interstitial fluid. Larger molecules and nutrients pass through the endothelium by transcytosis, a process regulated by specific transporters. Transport of macromolecules occurs through the transcellular pathway (transcytosis, through the cell body regulated by the cell membrane lipid bilayer) that is regulated, in part, by cytoskeleton proteins such as actin [24,25,26,27], and is often altered by immune activation. Transendothelial electrical resistance (TEER) is an index of current flow via the paracellular route (through the junctions between cells and regulated by junctional proteins) or via the transcellular route [28]. These different regulatory mechanisms may become more prominent depending on the phenomenon being studied. For example, we have shown that TNF-α activation had a larger impact on BBB permeability to a 40-kDa dextran (threefold increase) compared to TEER (23% reduction), indicating a shift from transcellular to paracellular transport [29]. Continuous capillaries exist in a more specialized state in the blood–brain barrier (BBB), where the EC are firmly linked together by tight junctions supported by pericytes and astrocytes, with no fenestrae and an extremely low rate of transcytosis. These special features protect the brain from harmful substances but are also obstacles for the delivery of therapeutic drugs into the brain [3].

Fenestrated capillaries have intracellular pores with a diaphragm of radially oriented fibrils that penetrate the endothelium, except in the EC of kidney glomerulus, which lacks a diaphragm [30,31]. PV1 (plasmalemmal vesicle associated protein-1) is a major structural component of the diaphragm’s formation [30,32,33]. The pores not only enable the rapid exchange of water but also permit the uptake and secretion of solutes between plasma and interstitial fluid. Fenestrated endothelia can be found in organs with absorption, filtration, and secretion functions, such as endocrine and exocrine glands, gastric and intestinal mucosa, choroid plexus, glomeruli, a subpopulation of renal tubules, and in some tumors. Though discontinuous endothelium is similar to fenestrated endothelium, its fenestrations have a larger diameter without a diaphragm and inadequate coverage by thinner basal lamina, which facilitates substance exchange and cell migration between blood and interstitial fluid. Sinusoids, as the discontinuous endothelium, are located in the liver, spleen, bone marrow, and several endocrine organs. Blood flow slows down in sinusoids to extend the duration time, allowing extensive exchange in these organs while the phagocytic cells patrol and engulf exogenous pathogens, damaged cells, and debris [3,34].

The glycocalyx, which consists of proteoglycans (mainly heparan sulfate) and glycoproteins, covers the membrane of endothelial cells, and also exhibits organ-specific differences. The glycocalyx regulates key vascular endothelium functions such as permeability, leukocyte adhesion, shear stress transmission, and anti-inflammatory defenses [35]. Glycocalyx size correlates to vessel diameter, ranging from several 100 nm in capillaries up to 10 µm in the carotid artery, thus directly affecting organ perfusion [36]. In the sinusoidal capillaries of the liver, the glycocalyx is thin, whereas, in the glomerular endothelium fenestrae, it provides an additional filtration barrier. In the continuous capillary, such as in the lung and cremaster muscle, the glycocalyx is thicker than it is in the liver. Amongst continuous endothelium, the glycocalyx in the brain microvasculature is particularly dense and resistant to inflammatory-induced vascular injury, and may serve a protective function [36].

The complement system is an important mediator of the immune system and is composed of 3 separate but overlapping pathways comprised of the classical, lectin, and alternative pathways. During inflammation or infection, complement components interact with EC, producing alterations in EC permeability, cytokine/chemokine production, leukocyte trafficking, and chemotaxis [37]. Variations in endothelial structure and function are observed at different levels of the vascular tree, resulting in differential responses to complement components. Comparative studies of renal and brain EC have demonstrated differential complement-mediated activation [38], with the kidney identified as an organ at risk for complement-induced EC damage [39]. Alternative complement pathway (AP) activation and regulation were compared in brain microvascular endothelial cells (BMVEC) with human glomerular vascular endothelial cells (GMVEC) to gain insight into the relative cerebral and renal injury in atypical hemolytic uremic syndrome (aHUS) and bone marrow transplantation-associated thrombotic microangiopathy (TA-TMA). BMVECs were more resistant to TNF-mediated AP activation than GMVECs [38]. Additionally, under both control and TNF-activated conditions, production of AP regulatory proteins was decreased in GMVECs compared to BMVECS, and they act to suppress the generation of C3a and C5a, which function as pro-inflammatory polypeptides (anaphylatoxins). These results suggest a mechanism of increased susceptibility of the kidneys and relative resistance of the brain to AP-mediated injury in aHUS and (TA-TMA) TA-TMA [38]. However, the differences in complement regulation in individual vascular beds are not completely understood [39].

### 2.2. Endothelium Heterogeneity across Species

Several studies have shown a number of differences and similarities in EC between different species [40,41,42,43,44]. These studies are important, as therapeutics for many diseases are often developed in animal models before clinical trials. However, there are concerns regarding the level of agreement between animal models and human diseases [45,46], and responses in mouse models often poorly correlate with the human conditions [46].

In vitro model cell systems have been used by several groups to investigate EC species differences [47,48]. Using a novel biomimetic microphysiological system (see description in Section 4.3), we examined human and mouse lung microvascular EC (HLMVEC and MLMVEC, respectively) under physiologically relevant flow conditions [47]. While a number of parameters were similar in the lung EC of mice and humans, significant differences were observed in microfilament alignment, TEER, and EC barrier permeability in response to inflammatory conditions, indicating differences in EC barrier function. On EC, selectins are important for leukocyte rolling, while adhesion molecules, such as ICAM-1 (intercellular adhesion molecule-1) and VCAM-1 (vascular cell adhesion molecule-1), are important for leukocyte transendothelial migration. In response to TNF, both HLMVEC and MLMVEC increased expression of E-selectin and VCAM-1. Surprisingly, in contrast to HLMVEC, ICAM-1, was not upregulated in TNF-activated MLMVEC. As ICAM-1 is an important regulator of leukocyte migration, our studies indicate that leukocyte–endothelial cell interaction may be regulated differently in mouse and human lung endothelia [47].

Other groups employing organ-on chip models have also demonstrated significant species differences. A novel liver-chip model, consisting of primary hepatocytes interfaced with liver sinusoidal EC, has shown different lipid accumulation in rat and human models following drug treatment [48]. Furthermore, the rat model was shown to develop indicators of liver fibrosis to a greater extent than the human model.

In addition to functional studies, protein expression studies have also been used to examine EC species differences. For example, expression of selenoprotein isolated from multiple different human umbilical vein endothelial cells (HUVEC) showed little variability and was similar over eight passages, while EC isolated from pig and bovine aorta showed significant differences in selenoprotein expression compared to human cells [49]. As discussed in Section 4, emerging techniques and biomimetic models of endothelial cell function will allow us to develop a clearer understanding of the significance of species differences between human and animal EC.

## 3. Endothelial Cell Alterations in Acute Inflammation

### 3.1. Endothelium Activation in Sepsis

**Figure 1:**
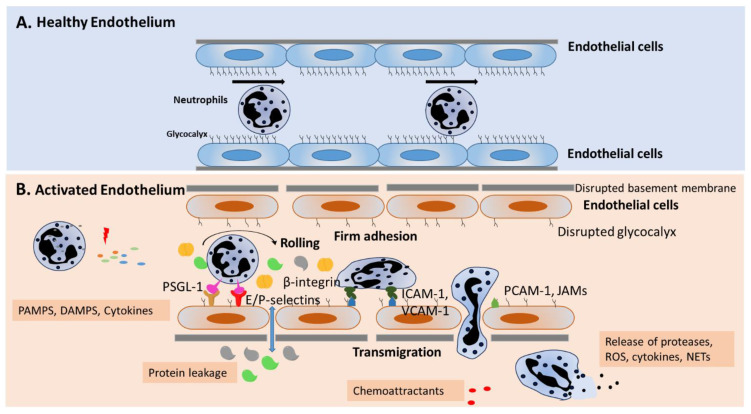
Endothelial cell activation in sepsis: increased barrier permeability and neutrophil migration: (**A**) Under normal conditions, the vascular endothelium is covered by the glycocalyx to form a tight barrier that regulates barrier permeability, neutrophil migration, and anti-inflammatory defenses. (**B**) During sepsis, PAMPS and DAMPS activate neutrophils and endothelial cells to produce cytokines and chemoattractants, which activate neutrophils to display surface molecules that interact with adhesion molecules expressed by activated endothelium. The rolling step involves interactions of E/P-selectin and their ligand (e.g., PSGL-1) on neutrophils, which slows down the neutrophil. The next step, firm adhesion, is mediated by adhesion molecules of endothelium, including ICAM-1, ICAM-2, and VCAM-1, and their neutrophil ligands, β_2_ integrins. In response to chemoattractants, adhered neutrophils migrate through endothelial junctions, involving PECAM-1 and JAMs. Activated neutrophils release cytokines, ROS, and proteases, or undergo NETs formation. During sepsis, the glycocalyx is degraded, endothelial cell tight junctions are damaged, and there is increased endothelial cell apoptosis, leading to damaged barrier function and increased permeability.

Sepsis is a clinical syndrome defined as life-threatening organ dysfunction caused by a dysregulated host response to infection that can be bacterial, fungal protozoal, or viral in origin [50]. The vascular endothelium is an active participant in the inflammatory response during sepsis (Figure 1). During inflammatory diseases such as sepsis, pathogen-associated molecular patterns (PAMPs) and damage-associated molecular patterns (DAMPs, cellular components released by damaged tissues) interact with pattern recognition receptors (PRR) located on immune cells and EC, and reprogram EC from an anti-inflammatory to a pro-inflammatory phenotype [51,52]. Signaling through PRR activates NF-κB and other proinflammatory transcription factors, resulting in the synthesis and release of cytokines (e.g., TNF-α and IL-1β), chemokines (e.g., IL-8 and MCP-1), and other pro-inflammatory signaling molecules, as well as the upregulation of adhesion molecules (e.g., VCAM-1, ICAM-1, and vascular endothelial cadherin (VE-cadherin) [53,54,55,56,57]. Sepsis-induced activation of EC results in alterations in barrier permeability, increased leukocyte–endothelial cell interaction, and induction of cell apoptosis. Adherens junction (cadherin family) and tight junction (occludin and claudin families) molecules are downregulated in the endothelium during sepsis [29,58,59,60,61,62]. Changes in barrier function are associated with shedding of the endothelial glycocalyx [51]. Loss of EC function induced by glycocalyx shedding in sepsis can lead to a dampening ability of EC to appropriately respond to shear stress. Glycocalyx shedding can also lead to impairment of EC antithrombotic fibrinolytic functions, resulting in thrombocyte adhesion and coagulation activation. As a result, activated EC release phosphatidylserine, which amplifies disseminated intravascular coagulation (DIC) [52,63]. The damaging of the glycocalyx also results in oxidative stress by compromising the antioxidant effects of heparan sulfate, which suppresses oxygen free radical production and maintains NO availability [64,65]. Following these events, there is increased permeability of proteins, fluids, and leukocytes, resulting in interstitial leakage or capillary leak syndrome, which could lead to organ damage.

During sepsis, an excessive number of neutrophils are recruited from blood vessels into the tissue compartment, leading to tissue damage and subsequent organ failure (Figure 1). Circulating neutrophils activated during sepsis are critical regulators of endothelial function by both secretion-dependent and adhesion-dependent events, which can damage EC, leading to organ dysfunction [66,67]. Neutrophils regulate EC through the formation of neutrophil extracellular traps (NETs), degranulation, and the secretion of reactive oxygen species (ROS) and reactive nitrogen species (RNS). The release of elastase, matrix metalloproteases, and myeloperoxidase (MPO) from neutrophil granules can cleave the protective glycocalyx, exposing the endothelial cell surface, resulting in enhanced leukocyte adhesion, endothelial cell activation, and increased barrier permeability [66,68]. The binding of NETs to vascular endothelium also increases permeability through disruption of adherens junctions and cytoskeleton reorganization [66]. Neutrophil-derived extracellular vesicles containing barrier disrupting cargo can also produce junctional disorganization, barrier disruption, and increased permeability [66]. Thus, while neutrophils are critical to host defense, neutrophil dysregulation in sepsis can play a critical role in the development of EC damage, leading to multiple organ dysfunction syndrome (MODS) and increased mortality.

### 3.2. The Classical Leukocyte Recruitment Process in Sepsis

The crosstalk between leukocytes and EC is composed of a series of interactions that orchestrate rolling, adhesion, and transmigration [55,69,70], leading to the migration of arrested leukocytes across endothelial cells to inflamed tissues via a multi-step process controlled by concurrent chemoattractant-dependent signals, adhesive events, and hemodynamic shear forces [57,71,72,73]. Selectins and a combination of integrins/immunoglobulins, chemokines, and chemoattractants mediate leukocyte–endothelium interactions [74]. The classical leukocyte recruitment process involves a multistep cascade including rolling, followed by firm adhesion and transmigration. Each step in the cascade is mediated by interactions of cell adhesion molecules on the surface of the endothelium with leukocyte ligands, as well as the expression of chemokines that direct the movement of leukocytes. Binding of endothelial E-selectin and P-selectin to leukocyte carbohydrate-based ligands initiates the rolling of leukocytes on the endothelium. Then, firm adhesion is achieved by binding of EC adhesion molecules, including VCAM-1 and ICAM-1, to leukocyte integrins. Leukocyte transmigration may occur between EC or through EC, known as transcellular and paracellular migration, respectively [75,76]. Transmigration is regulated by junctional adhesion molecules (JAM-C), VE-cadherin, and platelet endothelial adhesion molecule (PECAM-1) [77]. Similar to permeability, leukocyte migration occurs primarily in postcapillary venules supported by the preferential expression of E-selectin, P-selectin, VCAM-1, and ICAM-1 in these vessels [6]. However, as a result of organ-specific EC heterogeneity in cell structure and function, neutrophil trafficking to discrete organs may be differentially regulated.

### 3.3. The Classical Rules of Leukocyte–Endothelial Interactions Do Not Apply Universally

The classical leukocyte recruitment paradigm was primarily established by studying the microvasculature of the murine cremaster muscle [72]. However, further studies of different organ systems have determined that EC phenotype, morphology, and gene expression vary significantly among different vascular beds, resulting in different mechanisms of endothelial–leukocyte interactions. Leukocyte trafficking occurs primarily in postcapillary venules but, under certain conditions, it may also occur in other segments of the vascular tree, including large veins, arterioles, and capillaries [22]. For example, leukocyte adhesion and transmigration in the pulmonary circulation occurs chiefly in alveolar capillaries while, in the bronchial circulation, it occurs primarily in the post-capillary venules. Furthermore, the involvement of rolling motion or the requirement of E-/P-selectin in neutrophil recruitment seems to be dependent on the stimulus [77]. For example, leukocyte recruitment in Streptococcus pneumoniae-induced lung inflammation does not require the involvement of E- or P-selectin, while neutrophil recruitment in endotoxin-treated mice was dependent on E- and L-selectin [78,79]. Mechanical trapping of neutrophils in pulmonary capillaries may eliminate the need for rolling on the endothelium. As the diameter of the pulmonary capillaries is smaller than that of the neutrophil, neutrophils require longer time to deform to pass through the capillary. As a result, neutrophil transit time is prolonged, resulting in a higher concentration of neutrophils within the pulmonary capillary, known as neutrophil margination. Furthermore, neutrophils activated by cytokines become stiffer, contributing to neutrophil trapping in the capillary. Therefore, neutrophil capture in the lung is mediated by neutrophil deformability rather than selectin induced rolling [80,81]. However, prolonged firm adhesion and subsequent transmigration in the lung is at least partially dependent on ICAM-1 [82].

During liver inflammation, 80% of leukocyte adhesion occurs in the sinusoidal endothelium compared to 20% in post-sinusoidal venules [83]. Similar to the lung, the adhesion in the sinusoids is not dependent on selectins and thus rolling. However, it is worth noting that ICAM-1 is essential for leukocyte adhesion within the sinusoids [83]. In normal glomeruli, neutrophils and monocytes are retained in capillaries for up to several minutes (“dwell time”). Induction of glomerular inflammation results in activation of leukocytes and an increase in the duration of leukocyte retention in the glomerulus, which is shown to be Mac-1 dependent [84]. Though rolling is not required for leukocyte adhesion, the subsequent recruitment process in the glomeruli requires P-selectin and ICAM-1, as well as leukocytic PSGL-1 and β2-integrins [85]. However, it is noteworthy that glomeruli EC do not express P-selectin, but utilize platelets as a source of P-selectin for leukocyte recruitment [85].

### 3.4. Endothelium Activation in COVID-19

The coronavirus disease of 2019 (COVID-19) is caused by the severe acute respiratory syndrome coronavirus-2 (SARS-CoV-2) [86,87]. In severe cases of COVID-19, pneumonia can develop, resulting in viral sepsis. Early findings in this rapidly evolving field indicate that EC from different organs are involved in COVID-19, including the lung [88]. Angiotensin converting enzyme 2 (ACE2), by which the SARS-CoV-2 accesses the cell, is expressed in both lung EC and epithelial cells [89,90]. The entry of the SARS-CoV-2 virus into the cells is also mediated by other receptors including transmembrane serin protease 2, sialic acid receptors, and extracellular matrix metalloproteinase inducers, which are also expressed by EC [90]. There is both preclinical and clinical evidence supporting the hypothesis that the endothelium is a key target in COVID-19 [90]. Major symptoms found in COVID-19 patients, such as increased blood pressure, thrombosis, and pulmonary embolism, suggest that the SARS-CoV-2 virus targets the endothelium and alters endothelial function [90]. Furthermore, histopathologic features in COVID-19 suggest a complement-mediated endotheliopathy [91], and EC abnormalities were observed in many organs such as kidney, liver, lung, heart, and small bowel [88]. Additionally, evidence of the ACE2 expression on the vascular endothelium has led to the hypothesis that the endothelium is infected by SARS-CoV-2, inducing injury and activating complement, thus leading to a persistence in inflammation [92].

A major cause of morbidity and mortality of COVID-19 is the result of the development of acute lung injury, often leading to severe acute respiratory distress syndrome (ARDS) [93]. Pulmonary EC damage is an important contributing factor to the development of ARDS [94]. Lung pathology from COVID-19 patients demonstrated erosion of of endothelial cells in systemic venules and the presence of vasculitus, indicating strong vascular reactions [95]. In addition, there was evidence of significant neutrophil infiltration and trafficking into the alveolar space in these patients [95]. NETs have also been reported to be enhanced in COVID-19 patients compared to healthy controls [96]. NETS are important for pathogen clearance but can also significantly damage EC. Thus, based on preclinical and clinical studies, EC are thought to play a major role in the intiation and development of COVID-19, and targeting EC may provide novel therapeutic approaches for the treatment of this disease [94,97].

## 4. Emerging Techniques and Biomimetic Models to Study Endothelial Cell Function

### 4.1. RNA Sequencing

Emerging techniques, such as (single cell) RNA-sequencing, have facilitated the study of EC heterogeneity. RNA sequencing uses high-throughput sequencing methods to provide insight into the transcriptome [98,99] by revealing the differential gene expression patterns of EC from different species, different organs, and within the same tissue. For example, EC isolated from heart, lung, liver, and kidney have distinct expression patterns of gene clusters regulating barrier function, angiogenic potential, and metabolic rate. The heterogeneous organization of vasculature in various organs is characterized in part by the differential expression of EC markers, such as PECAM-1, VE Cadherin, and von Willebrand factor (vWF) [4]. Transcriptomics study with single cell RNA sequencing has shown a seamless continuum of transcriptional states of endothelial cells as they transition from arterial into capillary and then venous phenotypes (zonation), with the prevalence of BBB-associated trans-endothelial molecular transporters in capillaries and veins highlighting the known physiology at these vascular locations [100].

RNA sequencing has shown that, of the 18,910 genes expressed at baseline, the number of tissue-specific differentially expressed genes are 1692, 1052, 570 for brain, lung and heart endothelia, respectively, indicating different organ-specific gene signatures [101]. Lung endothelium has a high expression of genes involved in immune function such as leukocyte cell–cell adhesion, T cell activation, leukocyte migration, and regulation of immune system processes [101]. For example, high expression of MHC class II genes, which are responsible for antigen presentation, is observed in lung EC, implying its immune surveillance function [102,103]. Pulmonary endothelium, together with epithelial cells, form gas exchange units, which are in contact with the external environment and thus various external stimuli. The high expression of immune genes ensures a rapid immune response and emphasizes the important role of pulmonary endothelium in host defense. In accordance with the role of BBB in securing the brain microenvironment, brain EC express high level of genes involved in transport process [101,102]. A similar trend was found in heart EC, where the genes regulating cardiac muscle tissue development, myofibril assembly, and cardiac contraction were upregulated compared with EC from other organs [101].

### 4.2. Proteomics

The use of proteomics has provided important insight into differential expression of proteins within different types of EC. Compared to genomics, proteomics can provide direct insight into protein expression, allosteric regulation, alternative splicing, and dynamic protein–protein interactions [104,105]. Either an unbiased or targeted approach can be used to identify proteomic changes in disease pathologies or in response to varying experimental conditions. The unbiased approach focuses on identifying hundreds and thousands of proteins in a single analysis, while targeted proteomics requires prior knowledge of the proteins of interest before quantification of those proteins under different experimental conditions [105,106,107]. Protein analysis is usually accomplished by 2D gel electrophoresis (2DGE) for the separation of proteins based on charge (especially if proteins are post-translationally modified) and mass, and liquid chromatography in combination with mass spectrometry (LC-MS). For mass spectrometry-based proteomic analysis, matrix-assisted laser desorption/ionization (MALDI) and electrospray ionization (ESI) have become important tools for the ionization of biomolecules [108].

In organ/tissue-specific microvascular endothelial beds, not all omic changes are the same especially under normal or inflammatory conditions in human or mice EC. Although most studies of human EC proteomics have been conducted using HUVEC cells [109,110], the use of these cells does not represent an optimal model for recapitulating the 3D microenvironment of the microvasculature, which is significantly impacted under inflammatory conditions. More recent studies have employed mouse models and isolated mouse EC to examine EC heterogeneity under physiological and inflammatory conditions, particularly in brain, heart, kidneys, liver, and lung EC [101,111,112,113]. These studies demonstrated organ-specific EC have unique translatome patterns and omic signatures under homeostatic and inflammatory conditions. For example, in brain EC, top pathways included synapse organization, axon development, and neurotransmitter transport, while lung EC exhibited pathways correlating with immunity (i.e., T cell activation and leukocyte migration), as the lung is constantly exposed to inhaled pathogens. Conversely, heart EC have pathways related to cardiac muscle development and contraction due to energetic demands of cardiomyocytes. In summary, these murine studies demonstrate that organ-specific EC exhibit differential transcriptomic and proteomic expression, as well as unique cell surface markers; these are important considerations in the development of targeted therapeutics. Thus, the microvascular EC phenotype is affected not only by its unique, “zip code” omic signature but also by tissue microenvironment cues such as hemodynamic forces exerted by blood flow in a 3D morphology and interactions with nearby parenchymal cells in its microenvironment.

### 4.3. The Promise of Microphysiological Systems and Their Limitations

Microphysiological systems (MPS), a terminology often used synonymously with “organ-on-chip” and “microfluidic systems”, generally consist of an interconnected set of 2D and 3D cell cultures in microfluidic devices which can overcome many drawbacks of monolayer static cell culture systems by better reproducing the in vivo endothelial microenvironment. A strength of these systems is their ability to employ animal or human cells, which increases translatability and the potential for drug screening. MPS can serve as in vitro models of brain, GI tract, lung, liver, and skin microvasculature, and they are rapidly becoming the new standard for investigating vascular biology, pharmacology, and toxicology [29,48,114,115,116,117,118,119].

**Figure 2:**
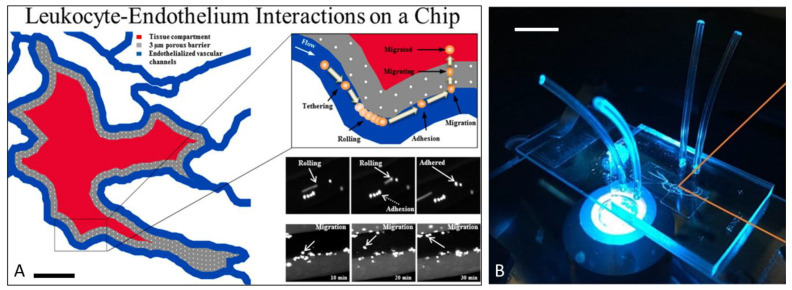
The biomimetic microfluidic assay (bMFA) mimics a physiologically relevant microvascular environment, which is used to study neutrophil-endothelial cell interactions: (**A**) The bMFA includes vascular channels and tissue compartment, which are connected through a 3 μm barrier region (scale bar 500 μm). (**B**) Microvascular network maps obtained in vivo are reproduced on PDMS to assemble the bMFA (scale bar 1 cm). ((**A**) Reproduced with permission from reference [127], https://pubs.acs.org/doi/10.1021/ac5018716, accessed on 25 May 2021. further permissions related to the material excerpted should be directed to the American Chemical Society. (**B**) Reproduced with permission from reference [140]).

**Figure 3:**
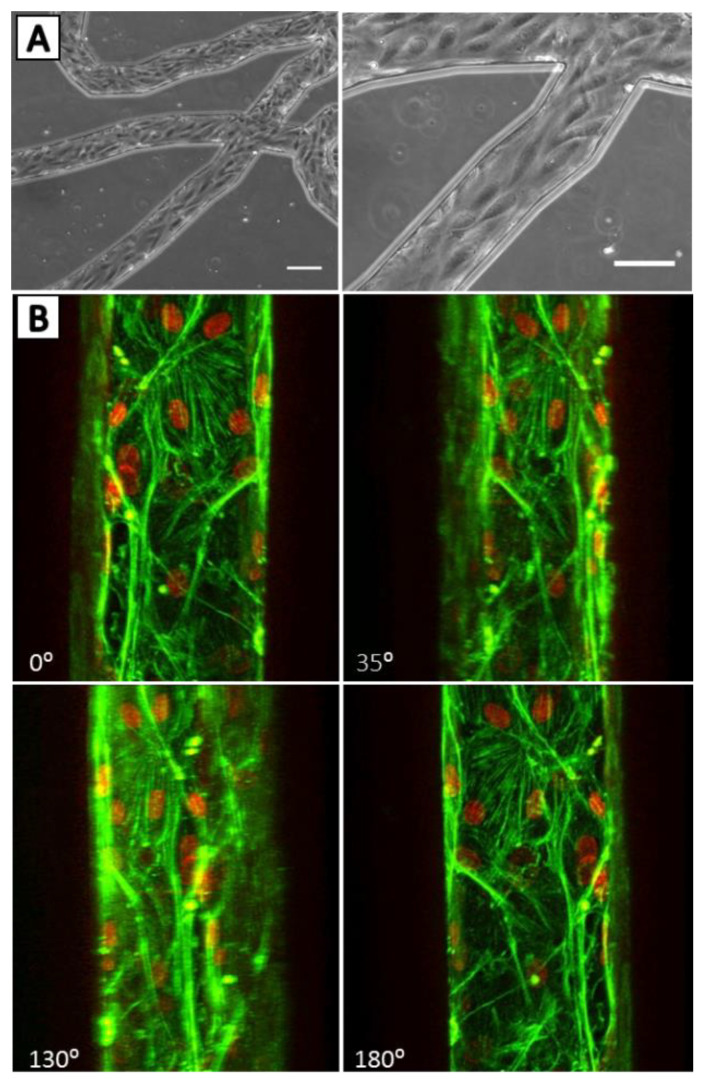
Endothelial cells form a complete lumen in the bMFA: (**A**) phase contrast images show endothelial cells are lined up in the direction of flow (scale bar is 100 µm). (**B**) Confocal micrograph of endothelial cells showing 3D lumen formation in the vascular channel; F-actin is labeled in green and nuclei is labeled in red. (Reproduced with permission from reference [120]).

Our group has developed a novel biomimetic microfluidic assay (bMFA) (Figure 2) [120], which reproduces the topography and flow conditions of the in vivo microvascular networks, for studying the leukocyte–endothelial cell interaction and endothelial function in vitro under physiologically realistic conditions (Supplementary Video). Microvascular network morphologies obtained from in vivo animal data were digitized using a Geographic Information System approach [121] for subsequent generation of synthetic microvascular networks using soft-lithography processes to develop the bMFA [122,123,124,125,126]. This microfluidic assay consists of vascular channels in communication with an extra-vascular tissue compartment filled with chemoattractants via a porous barrier region. Our bMFA resolves and facilitates real-time assessment of individual steps of neutrophil–endothelial cell interaction migration cascade in a single system, including rolling, firm arrest, spreading, and migration of neutrophils into the extra-vascular tissue space. EC form a confluent and complete 3D lumen in vascular channels under physiological flow (Figure 3). Neutrophils circulate in the vascular channels and interact with EC as they do under physiological conditions. The bMFA enables the assessment of individual steps of the entire neutrophil adhesion cascade in a single assay, including rolling, firm adhesion, and migration into the extravascular tissue space [47,120,123]. Neutrophil rolling and adhesion patterns to EC in the bMFA are similar to what is detected in vivo by intravital microscopy with preferential binding to activated EC near bifurcations [125,126,127]. Unlike what is observed with neutrophil binding to static EC monolayers, neutrophil binding is greatest in regions of low shear rates and minimal in regions of high shear rates (shear rate > 120 1/s), demonstrating that fluidic shear rate strongly influences neutrophil adhesion to EC [120].

Recently, additional biomimetic microphysiological systems have been developed that reconstitute the critical functional alveolar–capillary interface of the human lung by co-culture of pulmonary microvascular EC and epithelial cells subjected to cyclic mechanical strain, which can reproduce the complex integrated organ-level responses to bacteria and inflammatory cytokines introduced into the alveolar space, and model nanoparticle transport from alveoli into the lung vasculature [115]. Another microphysiological system, consisting of an air–liquid interface, with epithelial cells in the apical channel (exposed to air) and EC under shear flow in basolateral channels (exposed to plasma and/or blood cells), can model either small tubular airway or alveolar environments [116]. The latter system also enables functional imaging of the interior of the airway lumen (e.g., quantitation of cilia motion including beat frequency and mucociliary transport) and microvascular environment in the alveolus (e.g., surfactant production and leukocyte adhesion and migration) [116]. Similar in vitro airway models of the air–liquid interface have been used to study alveolar repair and inhalation toxicology [128,129,130]. The use of human cells may fill the gap regarding species heterogeneity between in vivo animal models and human disease. In addition, it has been shown that alveolar epithelial cells can be derived from human-induced pluripotent stem cells, implying the huge potential of these cells to overcome the problem of human cell availability [129]. A BBB on-a-chip microfluidic system enables the co-culture of brain EC with astrocytes under physiological flow conditions. The system is able to mimic the in vivo brain microenvironmental conditions and the optically clear microfluidic BBB on a chip allows for real-time visualization and measurements of BBB permeability and its dynamic interactions with leukocytes [29]. Several other microfluidic platforms modeling BBB degenerative diseases have also been developed recently [131].

While microphysiological systems are useful for providing important insight into EC function, like all other model systems, these have significant limitations, and the labeling of these systems as “organ-on-chip” should not be taken literally. For example, these systems usually include the co-culture of only a few cell types in a mostly mechanically stiff synthetic or gel scaffold, and they usually lack other important in vivo components such as other cell types present in the tissue and the matrix. Additional limitations include the type of polymer for chip design, time in culture, or exposure to proper levels of shear stress. For example, incompletely cured PDMS (the most common polymer used for chip design) can leach out and contaminate the medium and cells within the device, especially during long-term culture [132]. EC require exposure to shear flow to function normally and the level of shear to which they are exposed can determine their phenotype, as well as their morphology and function [133]. Design of microfluidic systems must include vascularized networks to provide oxygen and nutrients to multiple cell types that are required to mimic the in vivo microenvironment. The use of 3-D printing to create these more realistic MPS, especially for fabrication of circulatory and pulmonary systems, has been reported [134,135]. Furthermore, the use of iPSC (induced pluripotent stem cells) for creating vascularized organoids containing EC and pericytes that can assemble into capillary networks may represent a novel approach for studying diseases caused by vascular dysfunction [136]. More comprehensive reviews on the advantages and limitations of microfluidic models for various applications can be found elsewhere [132,137,138,139]. As with other model systems, MPS are most useful when employed to study specific, well-designed hypotheses leveraging the strengths of these systems.

## 5. Leveraging Emerging Technologies to Enhance Translatability of Ec-Targeted Therapeutics

Animal models, particularly rodents, have been used extensively for preclinical drug development. Unfortunately, as a result of significant species differences, the predictive power of success in human studies is severely limited. For example, all of the ~150 drugs that were recently developed in rodent models to treat sepsis have failed in clinical trials [141,142]. A significant limitation of rodent models is their differing and sometimes contradictory response to therapeutics compared humans [6,22,45,46,143,144]. Further, the phenotypic heterogeneity of different types of EC may also limit therapeutic development. This lack of clinical applicability is a significant limitation of rodent models for screening therapeutics for efficacy and toxicity for clinical applications [22,45,46,143,144]. Therefore, there is an urgent need for the development of models that better represent the human disease and new approaches to screen potential therapeutics. A recent NIH sepsis report encourages the “use of discovery science, computational, as well as cell-culture and organoid-type methods in preclinical sepsis research and organoid-type methods in practical sepsis research” using “human clinical material” and “preclinical research endpoints that specifically align with drug discovery” [145]. The microphysiological systems discussed in Section 4.3 can use human cells to significantly enhance translatability of therapeutic findings from animal models. These 3D culture systems can mimic the microenvironment of different organs, are not limited to a single cell types, and can model the communication of these multiple cell types, which is critical to organ function. In addition, the effect of therapeutics can be tested under physiologically relevant conditions, such as shear force for vascular modeling or mechanical stretch for lung modeling. EC have an important role in these microphysiological or organ-on-chip models, as they play a critical role in organ communication with the vascular system and drug delivery. These microphysiological systems have been used to study drug development and microvascular physiology in vascular networks, blood–brain barrier, and lung-on-a-chip [146,147,148]. In most cases, these models employ organs specific human EC to increase translatability.

We have utilized our bMFA system to test a novel therapeutic that targets lung inflammation. We identified protein kinase C-delta (PKCδ) as a critical regulator of inflammation. In a rodent model of sepsis, we demonstrated that PKCδ was activated in the lung endothelium, and selective PKCδ inhibition with a specific PKCδ-TAT peptide inhibitor attenuated neutrophil influx into the lung, decreased ICAM-1 and VCAM-1 expression, reduced alveolar-capillary permeability, decreased pulmonary edema, and preserved lung architecture [149,150,151,152]. These rodent studies suggest that targeting PKCδ is a potential therapeutic strategy in sepsis for the protection of organ function through the preservation of endothelial barrier permeability and the control of neutrophil migration. Using the bMFA, we were able to investigate specific mechanisms or steps in neutrophil–endothelial interactions that were regulated by PKCδ, and to ascertain whether PKCδ was a critical regulator of the inflammatory response in human cells controlling neutrophil infiltration across endothelia and a loss of barrier function [29,47,120].

**Figure 4:**
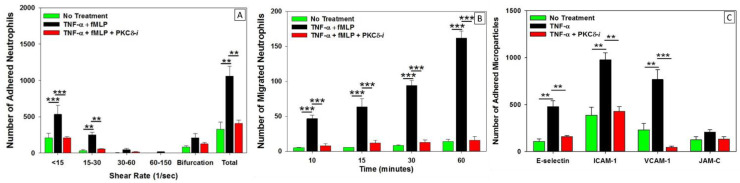
In vitro neutrophil migration in response to inflammation: regulation by PKCδ: (**A**) TNF-α treatment significantly increased human neutrophil adhesion to human pulmonary microvascular endothelial cells, which was inhibited following treatment with the PKCδ inhibitor. Neutrophil adhesion occurred preferentially at low shear rates and near bifurcations in the bMFA. (**B**) In response to fMLP, human neutrophil migration across TNFα-activated endothelial cells was significantly increased compared to untreated cells. Treatment with the PKCδ inhibitor reduced migration to untreated levels. (**C**) TNFα treatment increased the expression of the adhesion molecules, E-selectin, ICAM-1, and VCAM-1, but not JAM-C, indicating selective regulation. PKCδ inhibitor treatment significantly decreased E-selectin, ICAM-1, and VCAM-1, but not JAM-C expression. (Reproduced with permission from reference [47]) (*n* = 4 for panel A and B, *n* = 3 for panel C, data is presented as Mean ± SEM, one-way ANOVA, ** *p* < 0.01, and *** *p* < 0.001).

Our studies in bMFA demonstrate that proinflammatory cytokines, such as TNF, increase EC adhesion molecule expression and neutrophil trafficking through human endothelial cells [47,120,140,153]. Cytokine-activated human EC treated with the PKCδ inhibitor exhibited significantly decreased neutrophil adhesion and migration, consistent with our in vivo observations in rodents (Figure 4) [47,120,140]. Mechanistic studies demonstrated that PKCδ regulated expression of the adhesion molecules E-selectin, VCAM-1, and ICAM-1 (Figure 4) [47]. PKCδ is also an important regulator of endothelial cell permeability, and PKCδ inhibition attenuated TNF-mediated endothelial cell permeability and decreased TEER [29,47].

Microphysiological systems that mimic the microenvironment of the BBB show great potential for developing and screening therapeutics for treating neuro-inflammation [29,154]. For example, we employed our novel blood–brain barrier (BBB) on a chip (B^3^C), which uses primary human brain microvascular endothelial cells, to show the efficacy of the PKCδ inhibitor for treating different types of activated EC [29]. Similar to our rodent model of sepsis-induced brain inflammation, we demonstrated that PKCδ activation is a key signaling event that dysregulates the structural and functional integrity of BBB, which leads to vascular damage and inflammation-induced tissue damage due to neutrophil transmigration [29]. PKCδ inhibition prevented activation of human brain endothelial cells, protected BBB structure integrity, and prevented neutrophil migration across BBB endothelial cells. Our data suggest that PKCδ-TAT peptide inhibitor has therapeutic potential for the prevention or reduction of cerebrovascular injury in sepsis-induced vascular damage. Thus, PKCδ plays a key role in the regulation of proinflammatory signaling, controlling the activation and recruitment of neutrophils, as well as regulating endothelial permeability, TEER, and tight junction protein expression [29,120,149,151,152,155,156].

Other groups have employed similar microphysiological models to test the efficacy of potential therapeutics. Vascular network models have been used to investigate drug toxicities, the regulation of angiogenesis, and risk factors for the development of thrombosis [146]. A “vessel chip”, which contained a layer of confluent EC, was perfused with human blood at physiological relevant shear stress to examine blood–endothelial cell interactions [157]. This model was used to examine key risk factors for the development of thrombosis, such as platelet aggregation, platelet–endothelial cell interaction, clot formation, and markers of coagulation in response to antibodies against CD40L. Another model of vascular endothelium examined the regulation of angiogenesis and mechanisms involved in capillary vessel sprouting [158]. This microphysiological system was used to screen combinations of pro- and anti-angiogenic compounds to identify key mechanisms involved in the complex multicellular process of angiogenesis. Mimetic 3D models of the BBB have also been used to examine neuroinflammation and the impact on brain endothelial barrier permeability [159]. When brain microvascular endothelial cells were co-cultured with astrocytes and pericytes to model the BBB, the addition of TNF compromised barrier function and increased permeability, as demonstrated by decreased TEER and a reduction in the expression of tight junction proteins. Treatment with the glucocorticoid dexamethasone was protective, and it attenuated TNF-induced barrier permeability.

Several groups have used “lung-on-a-chip” to examine different pulmonary pathologies. These models contain both endothelial and epithelial cells to mimic the capillary–alveolar interface, often with cyclic mechanical stretching, to model lung movement during respiration [147]. These models have been used to examine the therapeutic efficacy of angiopoietin-1 and a transient receptor potential vanilloid 4 (TRPV4) ion channel inhibitor on IL-2- induced pulmonary edema [160]. Other studies have examined drug toxicity of the antitumor drug gefitinib, which targets epidermal growth factor receptor (EGFR), and the effects of titanium oxide and zinc oxide nanoparticles on endothelial and epithelial apoptosis [161,162]. We recently developed a biomimetic microfluidic tumor microenvironment (bMTM) model comprised of a co-culture of tumor and human breast tumor-associated EC in a 3D microenvironment [163]. Using this model, we demonstrated that EC permeability significantly increased in the presence of either tumor cell-conditioned media or tumor cells. The magnitude of this increase in permeability was significantly higher in the presence of metastatic breast tumor cells compared to non-metastatic ones. Thus, these emerging microphysiological systems, employing primary human EC to increase translatability, have significant potential in applications such as cell–cell/cell–drug carrier interaction studies and rapid screening of novel therapeutics/drug carriers.

## 6. Conclusions

EC are recognized as critical mediators in the progression of systemic inflammatory disease. Dysregulated endothelium leads to impaired functions, including upregulated expression of adhesion molecules, proinflammatory signaling, disrupted barrier integrity, and excessive neutrophil recruitment. However, the development of clinically relevant therapeutics has been complicated in large part by the recognition of EC heterogeneity between different species and different organs. The increasing utilization of emerging models such as microphysiological systems using human cells and other analytic tools may provide novel insights to not only better understand EC function but also to develop more clinically relevant novel therapeutics for treating inflammatory disease.

## Figures and Tables

**Table 1:** Examples of endothelial cell heterogeneity across organs.

EC Phenotypes	Location	Characteristics	Specialized Function	Diagram
Continuous	BBB EC of brain	Specialized tight junctions (occludin, claudin, and junctional adhesion molecule), supported by pericytes and astrocytes No fenestrae Extremely low rate of transcytosis Dense endothelial glycocalyx	Highly selective barrier to protect brain from harmful substances Specialized for efficient glucose transport to the brain Substrate-specific transporters for selective transfer (nutrients and metabolites)	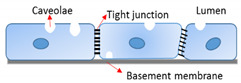
Fenestrated	Glomerular EC of kidney Endocrine and exocrine glands Gastric and intestinal mucosa	Intracellular pores with a diaphragm that penetrate the endothelium Rapid exchange of water Uptake and secretion of solutes Thick endothelial glycocalyx	Absorption, filtration, and secretion	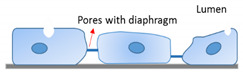
Discontinuous	Sinusoidal EC of liver, spleen, and bone marrow	Fenestrated with larger pore size Non-diaphragmed Thin and non-organized basal lamina Thin endothelial glycocalyx	Exchange of large solutes between plasma and interstitial environment Extensive exchange rate Dynamic filter for fluids, solutes, and particles Hepatic immune tolerance	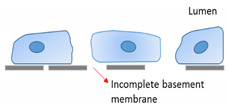

## Supplementary Materials

The following are available online at https://www.mdpi.com/article/10.3390/ijms22157770/s1.

## Abbreviations

EC	Endothelial cells
VEGF	Vascular endothelial growth factor
VEGFR2	VEGF receptor 2
BBB	Blood–brain barrier
HLMVEC	Human lung microvascular endothelial cells
MLMVEC	Mouse lung microvascular endothelial cells
TEER	Transendothelial electrical resistance
ICAM-1	Intercellular adhesion molecule-1
VCAM-1	Vascular cell adhesion molecule-1
HUVEC	Human umbilical vein endothelial cells
PAMPs	Pathogen-associated molecular patterns
DAMPs	Damage-associated molecular patterns
PRR	Pattern recognition receptors
VE-cadherin	Vascular endothelial cadherin
DIC	Disseminated intravascular coagulation
NETs	Neutrophil extracellular traps
ROS	Reactive oxygen species
RNS	Reactive nitrogen species
MPO	Myeloperoxidase
MODS	Multiple organ dysfunction syndrome
JAM	Junctional adhesion molecules
PECAM	Platelet endothelial adhesion molecule
COVID-19	Coronavirus disease of 2019
SARS-CoV-2	Severe acute respiratory syndrome coronavirus-2
ACE-2	Angiotensin converting enzyme 2
ARDS	Acute respiratory distress syndrome
vWF	von Willebrand factor
2DGE	2D gel electrophoresis
LC-MS	Liquid chromatography in combination with mass spectrometry
MALDI	Matrix-assisted laser desorption/ionization
ESI	Electrospray ionization
MPS	Microphysiological systems
bMFA	Biomimetic microfluidic assay
PKCδ	Protein Kinase C-delta
B^3^C	Blood–brain barrier on-a-chip
EGFR	Epidermal growth factor receptor
bMTM	Biomimetic microfluidic tumor microenvironment
